# Trainee-Reported Outcomes of Otology Simulation Training: Examining Effectiveness of the VOXEL-MAN Temporal Bone Simulator as a Training Adjunct in Mastoid Surgery

**DOI:** 10.7759/cureus.77360

**Published:** 2025-01-13

**Authors:** Lauren Bolton, Emma Gill, Savan Shah, Jaydip Ray, Gaurav Chawdhary

**Affiliations:** 1 ENT, Leeds General Infirmary, Leeds Teaching Hospitals NHS Trust, Leeds, GBR; 2 General Surgery, Auckland City Hospital, Auckland, NZL; 3 ENT, Sheffield Teaching Hospitals NHS Foundation Trust, Sheffield, GBR

**Keywords:** coronavirus, medical education, middle ear surgery, postgraduate education, training

## Abstract

Introduction: Mastoid surgery is technically challenging with complex three-dimensional (3D) anatomy. With reduced access to traditional training methods and increasing new technologies available, virtual reality simulation is being evaluated in a regional training program as an adjunct to existing training structures.

Methods: Twenty higher surgical trainees were instructed to complete a cortical mastoidectomy and posterior tympanotomy on a VOXEL-MAN simulator (VOXEL-MAN, Hamburg, Germany). A pre- and post-session questionnaire was completed.

Results: The visual analogue score for familiarity with the temporal bone 3D anatomy, cortical mastoidectomy and posterior tympanotomy surgical landmarks increased by 1.8 cm (p<0.0001), 1.4 cm (p<0.0001) and 2.2 cm (p<0.0001), respectively. Thirty percent of trainees felt that they had received adequate otology exposure within the last two years; 20% felt they would acquire enough experience to become a consultant otologist.

Conclusion: The VOXEL-MAN temporal bone simulator is effective in improving perceived anatomical and surgical knowledge and trainee confidence. Incorporating it into training programmes can help trainees compensate for the impact of the pandemic.

## Introduction

Learning to perform mastoid surgery is technically difficult and requires an excellent understanding of the three-dimensional (3D) anatomy of the temporal bone. Currently, most Ear, Nose and Throat (ENT) surgery trainees use cadaveric temporal bones when learning to operate [1]. However, the reduced accessibility and cost of these resources are significant drawbacks of this training modality. The COVID-19 pandemic has had a big impact on surgical training. The capacity of operating theatres, especially in ENT, was significantly reduced during the pandemic, as most procedures were elective and considered aerosol-generating. This trend has persisted, largely driven by ongoing resource and workforce shortages within the NHS [2]. This has meant that ENT surgical trainees have struggled to get sufficient operating theatre experience to develop surgical skills [3]. 

With the advancement of technology, there is now a range of temporal bone simulators validated for use in training [4-8]. The most extensively validated and the first commercially available temporal bone simulator is VOXEL-MAN (VOXEL-MAN, Hamburg, Germany). This is a virtual reality (VR) simulator that offers 3D visual and haptic feedback to perform mastoid surgery including drilling, dissecting, and navigating delicate structures like the facial nerve and otic capsule [4]. It has demonstrated face and content validity and there is evidence that it improves surgical training by improving the time to complete surgical tasks and reducing structural damage [9-14]. 

There are several benefits of using a virtual simulator in training; it allows extensive repetition of drilling with a simple click of the mouse, bypassing the cost and time implications of setting up new temporal bones in alternate training settings. Furthermore, the anatomy can be adjusted to allow variations in difficulty, for example, a more sclerotic bone or low-hanging dura. Simulation training, in conjunction with traditional methods, has the potential to significantly enhance the training of surgeons in this complex anatomical region [4]. While there is existing evidence that simulation training can improve certain surgical metrics, current literature has not considered the impact of simulators on trainee confidence or perception of their ability to perform said procedures or examined the use of training simulators within a wider training programme. Therefore, the objectives of the study were to evaluate trainee-reported outcomes on the use of the VOXEL-MAN temporal bone simulator within a regional training scheme for higher surgical trainees. In doing so, we hope to highlight the effectiveness of VR simulators in addressing the gap created by missed training opportunities and to better equip otolaryngology trainees with the essential anatomical knowledge and technical skills prerequisite to entering the operating theatre.

## Materials and methods

All ENT higher-surgical trainees within the Yorkshire and Humber training deanery in England were invited to participate in the study. Twenty trainees from CT2 to ST8 accepted the invitation to attend a session using the VOXEL-MAN simulator. Each participant had an individual session and was given an orientation to the simulator by a consultant otologist. In addition, the simulator manual was provided for troubleshooting. Junior trainees with less operative experience were shown how to undertake a cortical mastoidectomy and posterior tympanotomy. Trainees were then given three hours of independent simulator training to complete unlimited temporal bone dissections, including cortical mastoidectomy and posterior tympanotomy, and encouraged to explore further dissections. Trainees were provided with an automated feedback score from the simulator and had a debrief at the end of the session with the same consultant otologist. A pre- and post-session questionnaire was used to evaluate the outcome measures. These were measured using a visual analogue score (VAS) which is a method of recording a psychometric response to characteristics of a subjective nature [15]. VAS requires participants to place a cross on a 100mm line in response to a statement. The distance from the start of the line to the centre of the cross was measured and the score obtained to the nearest millimetre. The difference between the pre- and post-questionnaire scores was then calculated. The questionnaire is included in the Appendix. 

Main outcome measures

Six self-reported outcome measures were tested. These were: familiarity with the use of drill; familiarity with temporal bone 3D anatomy; familiarity with the surgical landmarks for posterior tympanotomy and cortical mastoidectomy; and trainee’s self-perceived level of confidence in both procedures. Data was also collected on trainees’ otology training in their real-world practices. 

Statistical testing

The difference in the VAS score for familiarity with temporal bone 3D anatomy, cortical mastoidectomy and posterior tympanotomy pre- and post-session was determined by a two-way ANOVA. The difference in the VAS score for the use of an otological drill, confidence in performing cortical mastoidectomy, and confidence in performing posterior tympanotomy pre- and post-session was determined using a paired t-test. 

## Results

The study was composed of 20 ENT trainees ranging from the grade of CT1 to ST8 (Table 1). Thirteen of these trainees were using the simulator for the first time, while seven had prior experience and had used the simulator at least once prior to the study.

**Table 1:** Distribution of trainees undertaking the training and questionnaire. There were 20 trainees in total. CT: Core Trainee; ST: Specialty Trainee

Grade	N	%
CT2	1	5%
ST3	3	15%
ST4	5	25%
ST5	3	15%
ST6	3	15%
ST7	4	20%
ST8	1	5%

**Figure 1:**
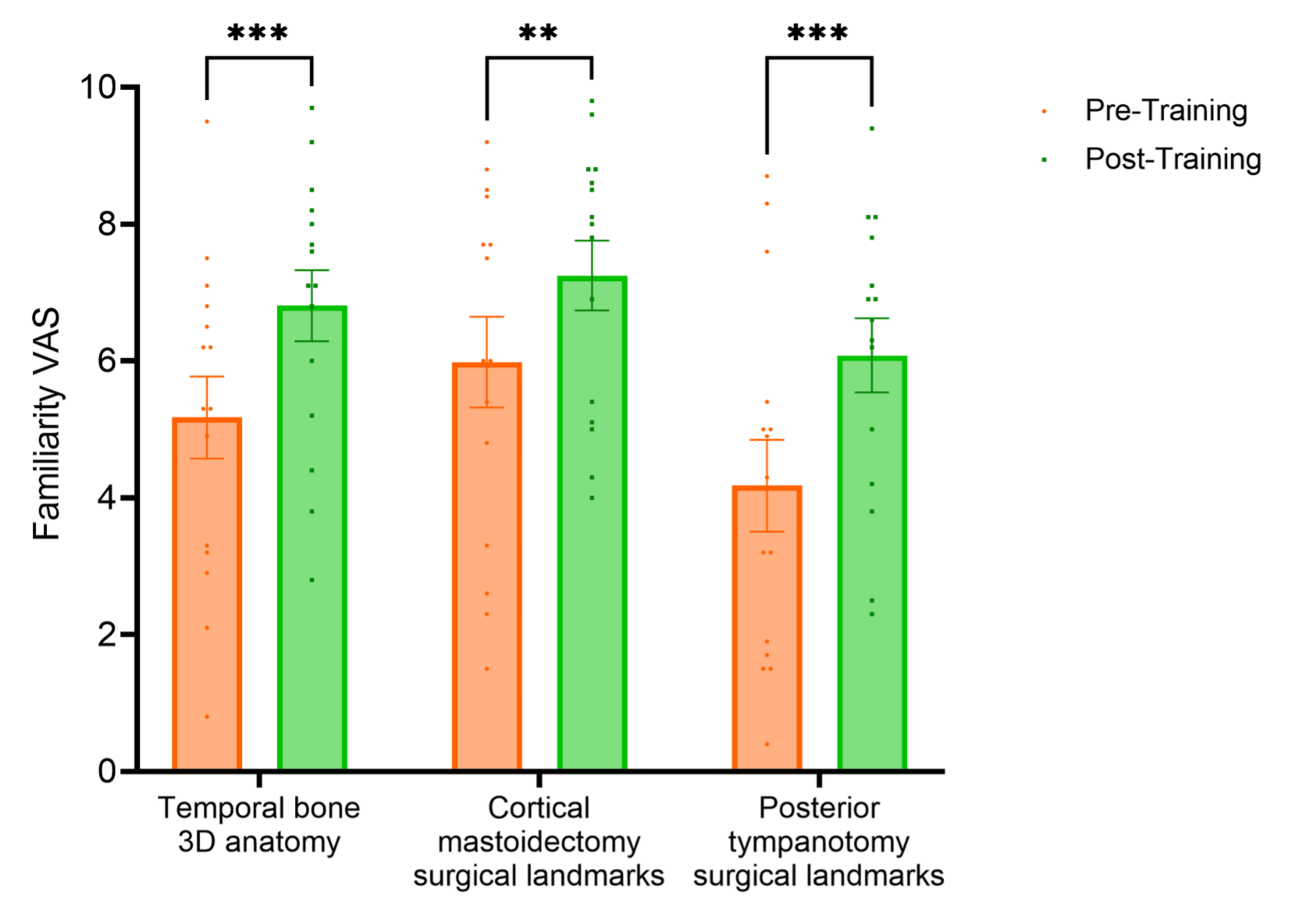
Graph showing familiarity with temporal bone anatomy, cortical mastoidectomy and posterior tympanotomy. The VAS score for familiarity with temporal bone 3D anatomy, cortical mastoidectomy and posterior tympanotomy surgical landmarks increased by 1.8 cm (p<0.0001), 1.4 cm (p<0.0001) and 2.2 cm (p<0.0001) respectively from pre-session to post-session. Statistical significance was determined by a two-way ANOVA. VAS: Visual Analogue Score *The level of statistical significance is set as p < .05

**Figure 2:**
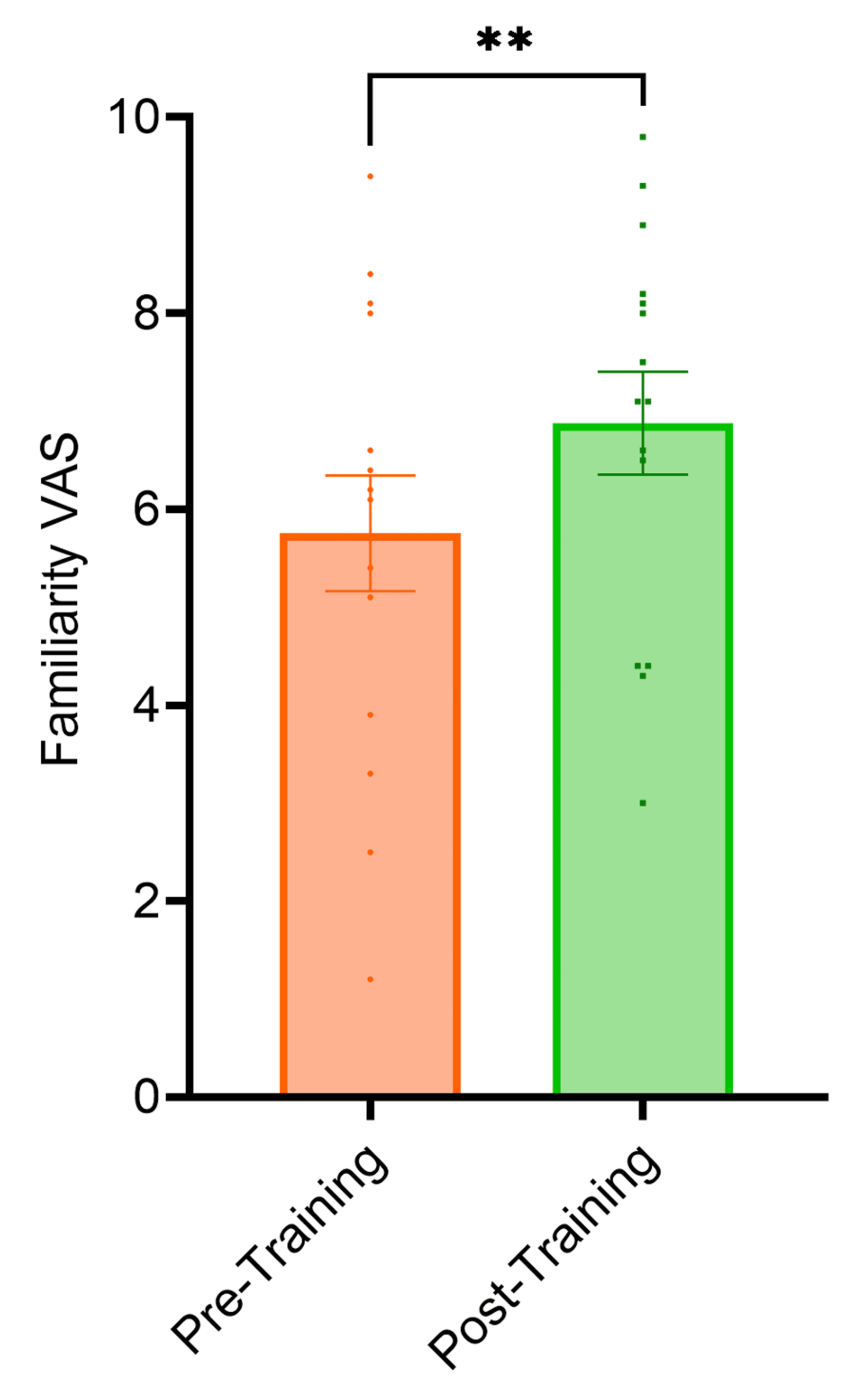
Graph showing familiarity with the use of the ontological drill. The VAS score for familiarity with the use of an otological drill increased by 1.5 cm (p<0.0001) from pre-session to post-session. Statistical significance was determined using paired t-tests VAS: Visual Analogue Score *The level of statistical significance is set as p < .05

**Figure 3:**
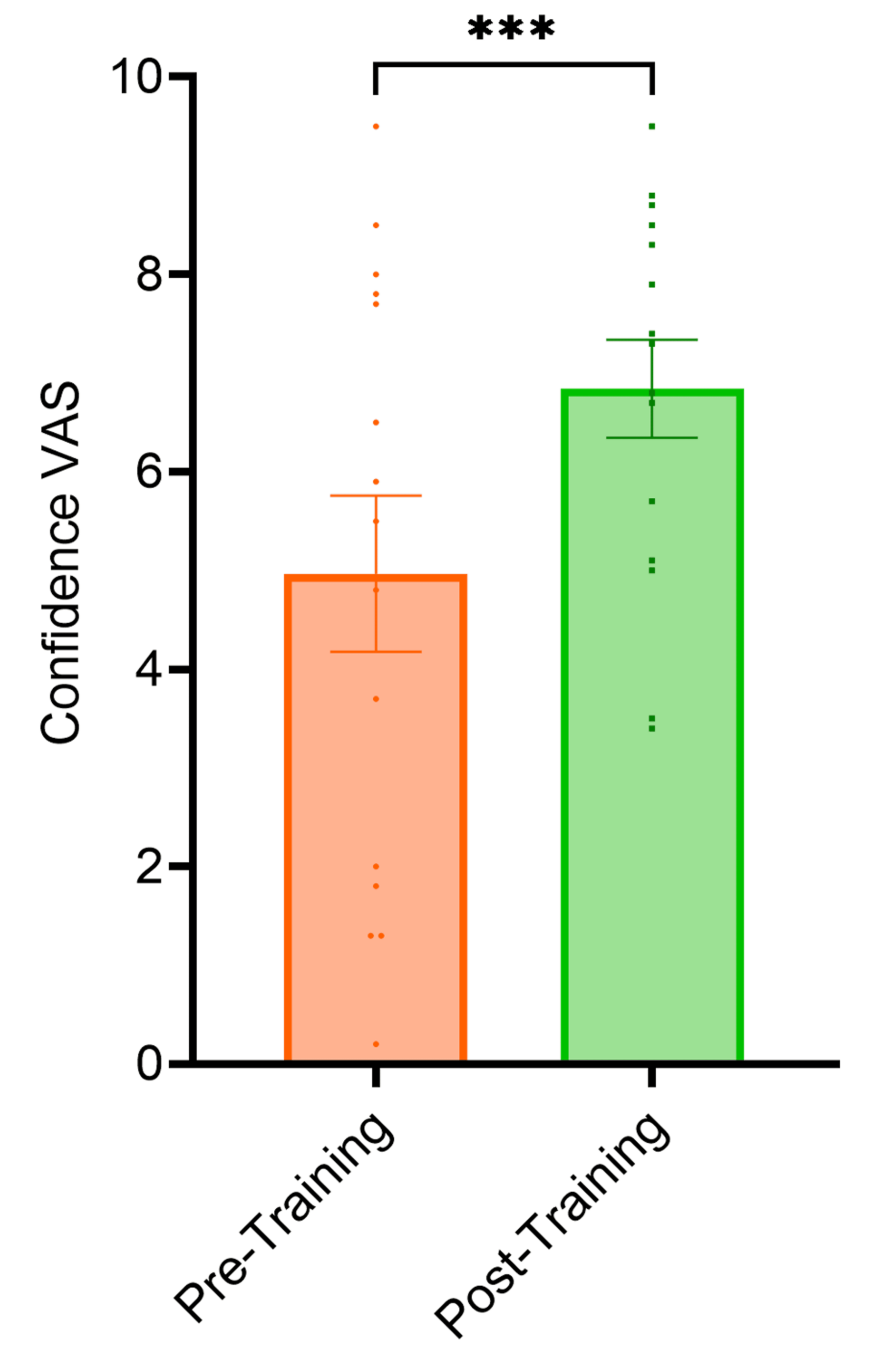
Graph showing confidence in performing cortical mastoidectomy pre- and post-training. The VAS score for confidence in performing cortical mastoidectomy increased by 2.1 cm (p<0.0001). Statistical significance was determined using paired t-tests VAS: Visual Analogue Score *The level of statistical significance is set as p < .05

**Figure 4:**
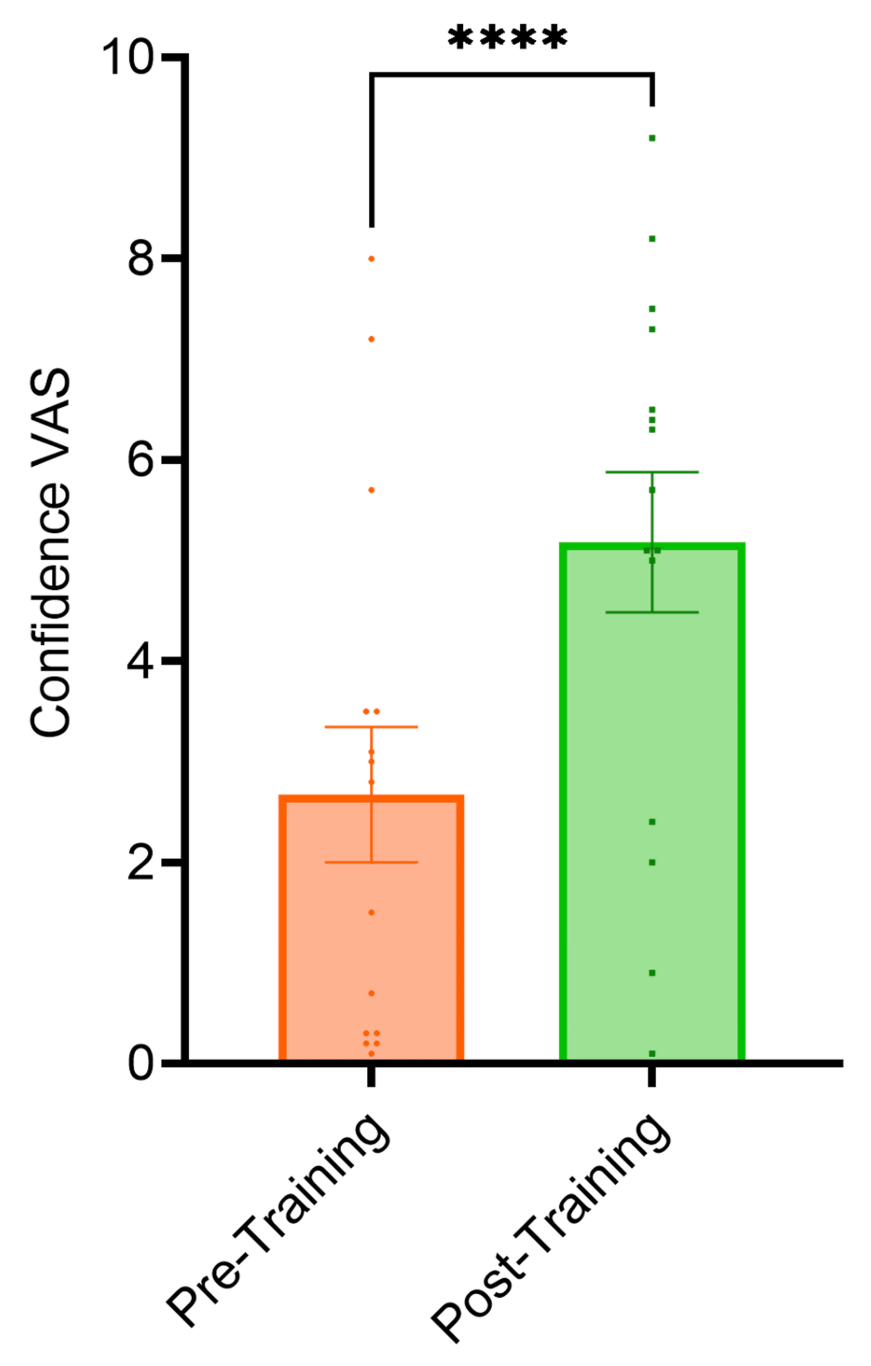
Graph showing confidence in performing posterior tympanotomy pre- and post-training. The VAS score for confidence performing posterior tympanotomy increased by 2.6 cm (p<0.0001). Statistical significance was determined using paired t-tests VAS: Visual Analogue Score *The level of statistical significance is set as p < .05

The VAS score for familiarity with temporal bone 3D anatomy, cortical mastoidectomy and posterior tympanotomy surgical landmarks increased by 1.8 cm (p<0.0001), 1.4 cm (p<0.0001) and 2.2 cm (p<0.0001) respectively from pre-session to post-session (Figure 1). The VAS score for familiarity with the use of an otological drill increased by 1.5 cm (p<0.0001) from pre-session to post-session (Figure 2). The VAS score for confidence performing cortical mastoidectomy increased by 2.1 cm (p<0.0001) (Figure 3) and the VAS score for confidence performing posterior tympanotomy increased by 2.6 cm (p<0.0001) from pre-training to post-training (Figure 4).

Only 30 % of trainees (n=6) felt that they had received adequate otology exposure within the last two years; 80% felt that they were on track to pass CCT; however, only 45 % felt they would receive enough exposure to be competent in otology and just 20% felt they would acquire enough experience to practise as an otologist. The main challenges reported by trainees were a lack of operating lists (especially during the COVID-19 pandemic), multiple trainees competing for available lists, lack of protected theatre time due to ward jobs/on-calls and a lack of uncomplicated cases.

In the post-training questionnaire, 95% of trainees described the simulation session as either “useful” or “very useful”. One hundred percent of trainees recommended simulation training at least once a year with 79% advising provision of sessions between two and four times per year. The most repeated positive feedback was that it was a useful tool for anatomy revision and that it improved familiarity with the use of the otological drill, this was particularly prevalent in the responses from more junior trainees. The main suggestion for improvement, as mentioned by three separate trainees, was including a structured session with supervision to allow for feedback and tutoring. This would run a fixed number of times during the year with the option for additional unsupervised sessions as per trainees' individual requirements. Others suggested the inclusion of more consultant supervision during the training as they felt the usefulness of the automated feedback was limited. Overall, most comments were positive and feedback from trainees was in favour of implementing simulation into the curriculum moving forward. 

## Discussion

In a post-COVID-19 era where surgical training and, in particular, otology training has been significantly negatively impacted [3], the effectiveness of new technology and training methods needs to be explored and evaluated to meet the educational needs of trainee surgeons and the population need for competent otologists. This paper demonstrates that the VOXEL-MAN temporal bone simulator can improve perceived knowledge and confidence in performing a cortical mastoidectomy and posterior tympanotomy, two hallmark steps in learning mastoid surgery. The overarching implications of our findings support the integration of VR simulation as a valuable tool in Otology training and offer a cost-effective solution to resource constraints in the NHS. 

This was evaluated on a regional level using a questionnaire incorporating the VAS, as well as qualitative feedback. The main outcomes of the study show that perceived knowledge of temporal bone 3D anatomy as well as surgical landmarks for cortical mastoidectomy and posterior tympanotomy improved after the session. Furthermore, confidence to perform these procedures also improved after the session. This finding is concordant with previous studies which have evaluated the VOXEL-MAN simulator [16]. Additional findings of the study show that only 30% of trainees felt that they had received adequate otology exposure within the last two years. This is aligned with the national quantitative data on experience in the UK [3]. Furthermore, only 20% of trainees felt that they were on track to have sufficient experience to work as a consultant otologist. This clearly demonstrates the urgent need to support trainees in innovative ways.

Whilst this study demonstrates perceived improvement in knowledge and confidence, one of the limitations of this work is that this data is subjective: it does not measure if the session is objectively improving the skill of the trainee. However, a key aim of surgical training programmes is to develop the trainee’s own familiarity and confidence with the anatomy, procedures, and equipment. It is a prerequisite for preparing the trainee to make effective use of real-world operating experience and to perform the actual procedure. In this respect, our study clearly demonstrates trainees’ improved familiarity and confidence level in a range of metrics, thereby supporting their training and preparing them for real-world performance.

Other papers have measured objective improvements in performance by tracking trainees’ automated feedback scores or by using direct trainer supervision with a validated scoring system [17,18]. Future studies should combine these objective measures with the trainee VAS to evaluate whether the trainees' perceived improvement correlates with real-world progression. 

In the future, we plan to use these results to inform our regional teaching program and to support trainees in developing their skills in mastoid surgery and making them aware of what they can expect in terms of improved confidence and familiarity by utilising simulation training. Simulation training programmes in otology are in their infancy and there is room to develop and mature the teaching methods used to integrate this modality with more traditional methods. Examples of this from other literature include creating step-wise and time-based exercises to focus on specific ‘set piece’ procedures in otology: skeletonization of sinodural angle, identification of lateral semicircular canal and short process of incus, delineation of facial nerve and chorda tympani and extended cortical mastoidectomy [19]. It may also be useful to pair the simulation sessions alongside regional cadaveric training, both for trainees’ enhanced learning and to better understand the relative merits and weaknesses of the two modalities, and how they might better complement each other. As the technology develops it will also be interesting to see if more advanced procedures can be taught such as stapedectomy or cochlear implant insertion, and whether simulation can model soft tissue work as well. Currently, VOXEL-MAN is just one of many temporal bone simulators, with several others available including CardinalSim [6] and Virtual Ear Surgery [8]. Future studies should further explore the efficacy of such simulators in preparing trainees for a broader range of complex surgical procedures. 

## Conclusions

This study has demonstrated that the VOXEL-MAN temporal bone simulator is effective in improving trainees’ familiarity and confidence in aspects of mastoid surgery, against the background of a post-pandemic disruption in surgical training opportunities. By incorporating state-of-the-art technology, such as the VOXEL-MAN temporal bone simulator into surgical training programmes, trainers can help trainees mitigate the loss of training opportunities that may have occurred.

## Appendices

Voxel-Man training programme - pre-session questionnaire

What ST or CT level are you?

How many times have you attended a Voxel-Man Simulator session in the past?

What is your current intended career direction?

Otology                                   Non-otology

In the last two years, what best describes the amount of direct otology operating experience in theatre you have received:

Inadequate amount

Adequate amount

Good amount

Excellent amount 

Do you feel you will acquire enough direct otology operative experience during training to:

Achieve CCT                                                                Yes                   No

Be competent in otological surgery                          Yes                   No

Practise as an otologist                                                          Yes                   No

What challenges do you face in obtaining enough otology operative experience? (please be as detailed as possible)

How many mastoids would you drill in a typical half-day list?

How many mastoids would you drill in a typical half-day temporal bone course?

Please indicate on the scale below your level of familiarity with:

Temporal bone 3D anatomy

Least familiar

Most familiar

Use of an otological drill

Least familiar

Most familiar

Cortical Mastoidectomy surgical landmarks

Least familiar

Most familiar

Posterior Tympanotomy surgical landmarks

Least familiar

Most familiar

Please indicate on the scale below how confident you feel in performing the following procedures:

Least confident

Most confident

Cortical Mastoidectomy

Posterior Tympanotomy

Least confident

Most confident

Voxel-Man training programme - post-training questionnaire

Please indicate on the scale below your level of familiarity with:

Temporal bone 3D anatomy

Least familiar

Most familiar

Use of an otological drill

Least familiar

Most familiar

Cortical mastoidectomy surgical landmarks

Least familiar

Most familiar

Posterior tympanotomy surgical landmarks

Least familiar

Most familiar

Please indicate on the scale below how confident you feel in performing the following procedures:

Cortical Mastoidectomy

Least confident

Most confident

Posterior Tympanotomy

Least confident

Most confident

How useful did you find your otology simulation training? (circle one)

Not at all useful

Not useful

Neutral

Useful

Very useful

In your opinion, how many times a year should simulation training in otological surgery be offered to surgeons-in-training?

Any other comments?
